# Fulminant Influenza Myocarditis Requiring Extracorporeal Membrane Oxygenation (ECMO) Support

**DOI:** 10.7759/cureus.83183

**Published:** 2025-04-29

**Authors:** Kenney Abraham, Phillip Key, Matthew C Pelletier, Ryan Heslin

**Affiliations:** 1 Internal Medicine, Stony Brook University Hospital, Stony Brook, USA; 2 Cardiology, Stony Brook University Hospital, Stony Brook, USA

**Keywords:** bivad, fulminant myocarditis, influenza, mcs, va ecmo

## Abstract

Viral infections may lead to myocarditis, which is inflammation of the myocardium. This inflammation, when severe enough, can result in left ventricular dysfunction and potentially reduce the left ventricular ejection fraction (LVEF). In rare cases, the effects of this inflammation lead to hemodynamic changes that can be life-threatening. We discuss a case of a 38-year-old female recently diagnosed with influenza A (H3 subtype) who presented to our institution’s emergency department for evaluation after an episode of syncope, as well as intermittent chest pressure and dyspnea on exertion. Initial vitals displayed a heart rate of 87 bpm and blood pressure of 105/66 mmHg. The physical examination demonstrated a regular rhythm, no lower extremity edema, and lungs that were clear to auscultation. She was found to have an elevated pro-B-type natriuretic peptide level of 6152 pg/mL and a positive influenza A polymerase chain reaction (PCR) test. A transthoracic echocardiogram (TTE) was obtained and demonstrated globally reduced left ventricular systolic function with an estimated ejection fraction of 28%, as well as reduced right ventricular systolic function.

Over the next six hours, the patient became progressively tachycardic and hypotensive, with a heart rate of 135 bpm and a blood pressure measured at 46/28 mmHg. She was initially admitted to the cardiovascular ICU and started on dobutamine and vasopressin. Pulmonary artery catheterization was completed for better evaluation of cardiogenic shock, and it demonstrated a severely reduced cardiac index of 0.9 L/min/m^2^. Due to concerns of worsening cardiogenic shock and impending circulatory collapse, mechanical circulatory support was initiated via veno-arterial extracorporeal membrane oxygenation (VA-ECMO), and she was admitted to the cardiothoracic surgery ICU. Several days later, a biventricular assist device (BiVAD) was implanted with the goal of discontinuing ECMO as a bridge to transplant. Shortly afterwards, a repeat echocardiogram demonstrated a normalized left and right ventricular systolic function, and the BiVAD was removed. Ten days after the initiation of ECMO, it was able to be discontinued, and the patient was decannulated. The patient was discharged home in stable condition. This case exemplifies how fulminant myocarditis (FM) can have positive outcomes, even in critically ill patients, when the timing of intervention is early and aggressive.

## Introduction

Myocarditis refers to the inflammation of the muscular layer of the heart, known as the myocardium [1]. This condition results from an injury to the cells of the myocardium (cardiomyocytes), and is most often caused by viral illness, but can also be due to autoimmune diseases or drug reactions [2]. This condition is commonly associated with symptoms such as chest pain, fever, and dyspnea [3]. However, in rare cases, these symptoms can evolve to a presentation that is much more life-threatening, referred to as fulminant myocarditis (FM). This condition can be defined as myocarditis that progresses acutely to severe symptoms of heart failure and hypotension within two weeks of onset [4]. In some cases, FM may lead to hemodynamic instability, requiring vasopressors or even mechanical circulatory support (MCS) [5]. 

We report a case of a 38-year-old female who presented to the emergency department after a syncopal episode, having tested positive for Influenza A/H3 four days prior. In most parts of the world, viral illnesses are the most common cause of myocarditis [6]. Although Coxsackie species subtype B is the virus most commonly associated with myocarditis, the influenza virus has also been implicated [7]. This case highlights how influenza can not only cause myocarditis but also facilitate a progression to FM, as well as the treatment modalities used to manage critically ill patients with this disease state. 

This article was previously presented as a meeting abstract at the 2024 New York Chapter Annual Scientific Meeting on October 26, 2024.

## Case presentation

A 38-year-old female without any relevant past medical history presented to the emergency department following an episode of syncope. Four days prior, she had been diagnosed with Influenza A/H3 as an outpatient and reported four days of upper-respiratory symptoms as well as intermittent chest pressure, with dyspnea on exertion. Initial vitals showed a heart rate of 87 bpm and a blood pressure of 105/66 mmHg. Although initially stable, the patient deteriorated with increasing tachycardia to 135 bpm and worsening hypotension, with a blood pressure of 46/28 mmHg. Blood work drawn in the emergency room was notable for a lactic acid level of 9.7, pro-B-type natriuretic peptide value of 6152 pg/mL, and a mildly elevated, but gradually up-trending high-sensitive troponin (Table 1).

**Table 1:** Lab results from the day of admission ALP: alkaline phosphatase; ALT: alanine aminotransferase; AST: aspartate aminotransferase; BUN: blood urea nitrogen; GFR: glomerular filtration rate; SGOT: serum glutamic-oxaloacetic transaminase; SGPT: serum glutamate-pyruvate transaminase

Lab test	Patient value	Normal range
Sodium	137	135 - 145 mmol/L
Potassium	4.0	3.5 - 5.0 mmol/L
Chloride	102	96 - 106 mmol/L
Bicarbonate	20	22 - 28 mmol/L
Glucose level	143	70 - 99 mg/dL (fasting)
BUN	10	6 - 20 mg/dL
Creatinine	0.93	0.6 - 1.2 mg/dL
GFR	81	>60 mL/min/1.73 m²
Anion gap	15	3 - 11 mEq/L
Calcium	8.2	8.5 - 10.2 mg/dL
Phosphorus	3.2	2.5 - 4.5 mg/dL
Magnesium	2.2	1.7 - 2.2 mg/dL
Bilirubin, total	0.2	0.1 - 1.2 mg/dL
Bilirubin, direct	<0.2	<0.3 mg/dL
ALT (SGPT)	41	7 - 56 U/L
AST (SGOT)	51	10 - 40 U/L
ALP	43	44 - 147 U/L
Albumin	3.6	3.5 - 5.0 g/dL
Cardiac troponin (high sensitivity)	62	<19 ng/L (varies by assay)
Lactic acid	9.7	0.5 - 2.2 mmol/L
Pro-B-type natriuretic peptide	6152	<125 pg/mL (age <75); <450 pg/mL (age >75)

**Figure 1:**
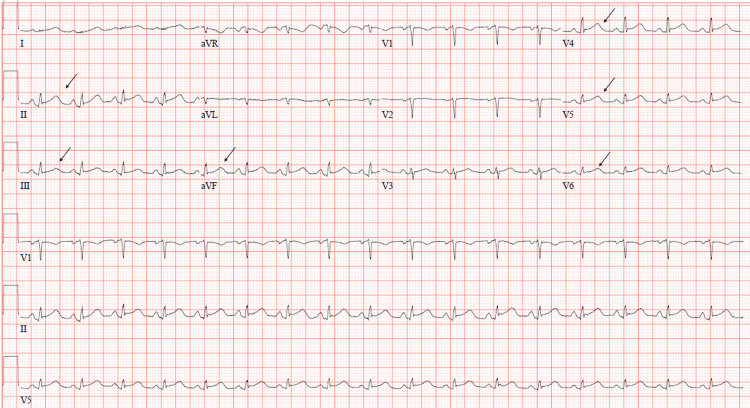
Initial EKG on admission showing ST-segment elevations in the inferior leads as well as V4-V6 (marked by arrows) EKG: electrocardiogram

EKG showed sinus tachycardia with ST-segment elevation in the inferior leads as well as V4-V6 (Figure 1). Transthoracic echocardiogram (TTE) showed severely reduced global left ventricular systolic function with an estimated left ventricular ejection fraction (LVEF) of 28% (obtained by Simpson's biplane method), consistent with FM (Video 1). As the patient was in cardiogenic shock, dobutamine and vasopressin were initiated. She was subsequently taken to the cardiac catheterization lab, and a pulmonary artery catheter was placed to allow for accurate measurements of hemodynamics, such as cardiac output, cardiac index, and systemic vascular resistance. Initial reading on the pulmonary artery catheter displayed a severely reduced thermodilution cardiac index at 0.9 L/min/m^2 ^(normal range: 2.5-4.0 L/min/m^2^). 

**Video 1:** Parasternal long axis on echocardiogram before the initiation of inotropic and mechanical circulatory support (LVEF: 25-30%) LVEF: left ventricular ejection fraction

The patient was intubated at this time as well due to worsening mental status and respiratory distress. An Impella device could not be placed due to difficulty with femoral and axillary arterial anatomy, and hence she was started on central veno-arterial extracorporeal membrane oxygenation (VA-ECMO) support. Due to ongoing difficulty with weaning ECMO support over a week, the patient required implantation of biventricular assist devices (BiVAD), which would be used as a bridge to transplant. Over the next few days, she required multiple blood transfusions and was found to have worsening renal failure, becoming oliguric and requiring initiation of continuous renal replacement therapy (CRRT). Despite initially requiring high doses of inotropes and vasopressors, her myocardial function gradually improved, as demonstrated by improving hemodynamic measurements on the pulmonary artery catheter and LVEF quantification on TTE (Video 2). VA-ECMO was then transitioned to veno-arterial-venous (VAV) configuration due to ongoing right ventricular dysfunction, but was later converted to veno-venous (VV)-ECMO for respiratory support as her LVEF normalized. As the patient became more stable over the next 10 days, the BiVAD was removed, VV-ECMO was successfully weaned off, and she was decannulated.

**Video 2:** Parasternal long axis view on echocardiogram after ECMO/BiVAD placement (LVEF: 60-70%) BiVAD: biventricular assist device; ECMO: extracorporeal membrane oxygenation; LVEF: left ventricular ejection fraction

She was subsequently extubated to a high-flow nasal cannula, with her supplemental oxygen requirements being titrated down as her condition improved. The patient was eventually weaned off of vasopressors and inotropes before she made a full recovery.

## Discussion

FM is a rare but deadly consequence of myocarditis. Its association with pathogens as common and preventable as the influenza virus emphasizes the importance of early recognition and intervention, while properly understanding its pathophysiology, epidemiology, and treatment guidelines. While the exact pathophysiology of FM is not fully understood, studies in the literature have traditionally classified its mechanism into three phases: viral, immune activation, and myopathy [8]. The initial insult to the myocardium begins with either the introduction of a new virus (like the influenza virus) or the reactivation of a dormant virus (like parvovirus B19), which utilizes myocytes as a host. This leads to the immune activation phase, which is characterized by inflammation and the activation of T cells, which neutralize antigens. Finally, the myopathy phase refers to the lasting, uncontrolled effects of the immune response, which can cause necrosis of cardiomyocytes, leading to ventricular remodeling and dilated cardiomyopathy over time [9].

Each year, approximately three to five million cases of severe influenza are observed worldwide, with up to 650,000 deaths reported by the World Health Organization (WHO) [10]. While myocarditis is considered an uncommon complication, prior studies have shown this association in approximately 5-10% of cases, although autopsy data suggest that myocardial involvement may be present in up to 40% of cases [11]. The discrepancy in these data highlights a possible underdiagnosis of myocardial injury as a result of influenza infections, suggesting that the true incidence may be greater than what is currently recognized.

While acute myocarditis and its progression to FM are both relatively uncommon conditions, the possibility of their occurrence should not be underestimated. According to the Global Burden of Disease registry, in 2019, the incidence of acute myocarditis in patients between the ages of 20 to 44 years was 6.1 per 100,000 in men and 4.4 per 100,000 in women. Of these patients, about 5-10% were reported to have progressed to FM [8]. With that said, the survival rate of patients with FM is between 50% and 70% overall, with a 59% survival rate with VA-ECMO [12].

Currently, treatment modalities that are effective in patients with FM involve methods of life support, consisting of MCS, respiratory support, and renal replacement [13]. Of these life support therapies, the most essential in the treatment of FM is MCS, which includes treatments such as intra-aortic balloon pumps (IABPs), Impella devices, or VA-ECMO, in addition to the initiation of inotropes and vasopressors. For patients who rapidly become hemodynamically unstable, it has been shown that IABP placement increases the survival rate by increasing blood pressure and decreasing cardiac afterload. Impella devices have also been found to improve survival rates by delivering blood from the LV to the aorta, thereby increasing perfusion to the rest of the body [14].

## Conclusions

Viral infections, including influenza, are the most common cause of acute myocarditis. In some patients, acute myocarditis can lead to global left ventricular dysfunction and rapid hemodynamic instability, otherwise known as FM. This disease state necessitates immediate intervention with the use of vasopressors and inotropes, and if it progresses, MCS may be indicated, which includes interventions such as VAD placement or VA-ECMO, as these methods enable life to be sustained in the setting of refractory cardiogenic shock. This report highlights the importance of early recognition and intervention to optimize outcomes in these patients.
